# Beirut Explosion Effects on COVID-19 Situation in Lebanon

**DOI:** 10.1017/dmp.2021.56

**Published:** 2021-02-16

**Authors:** Hashim Talib Hashim, Saad Uakkas, Abdallah Reda, Mustafa Ahmed Ramadhan, Morad Yaser Al Mostafa

**Affiliations:** 1 College of Medicine University of Baghdad, Al-Rasafa, Baghdad, Iraq; 2 Mohammed V University, Rabat, Morocco; 3 Carol Davila University of Medicine and Pharmacy, Faculty of General Medicine, Bucharest, Romania; 4 Princess Basma Hospital, Irbid, Jordan

**Keywords:** COVID-19, Beirut explosion, Lebanon, pandemics, disease outbreaks

Lebanon is an Arab country in the Levant region of Western Asia. With its population of 6.8 million people, it accommodated about 250 000 Palestinian refugees since the 1950s and 1.5 million Syrian refugees since 2010. Lebanon officially recognizes 18 religious communities within its population, with a Muslim and Christian majority.^1^

Lebanon reported the first case of coronavirus disease (COVID-19) on February 21, 2020: a 45-year-old woman returning from a pilgrimage to Qom, Iran, tested positive for the virus responsible for COVID-19, severe acute respiratory syndrome coronavirus 2 (SARS-CoV-2), and was admitted to a Beirut hospital. The government engaged in massive testing for suspects and initiated a national curfew, which helped limit the disease’s spread at the first 5 months with less than 100 daily cases on average.^2^ Since August, the country faced a surge in the COVID-19 cases number reaching around 1500–2000 daily cases in mid-December.^3^

On August 4, 2020, Beirut, Lebanon’s capital witnessed one of the most intense non-nuclear explosions in history. An estimated 2750 tons of ammonium nitrate that were deposited in the city port exploded shortly after 6:00 PM local time, causing at least 204 casualties, 6500 injuries, and US $15 billion in property damage, leaving an estimated 300 000 people homeless. The explosion was felt in neighboring countries, including Turkey, Syria, Israel, Palestine, and areas of Europe. It was detected as a seismic phenomenon of magnitude 3.3 by the United States Geological Survey.^4^

Following this, the country’s hospitals were filling up with hundreds of injured patients. Health care workers engaged in their full capacity to cope with the crisis situation. Multiple facilities got destroyed and numerous patients, including COVID-19 cases, had to be transported to the remaining functional structures. With hospitals and health workers being oversaturated dealing with the blast casualties, COVID-19-suspected people had less access to health care services.^4^

With only 2 psychiatrists per 100 000 people, the country was facing the pandemic and a deteriorating socioeconomic situation negatively affecting its population’s mental health. After the explosion, the situation got worse with a soaring number of calls for helplines and cases of mental illnesses.^5^ Such outcomes can have unfavorable effects on people’s COVID-19 risk perception levels and their respect of health guidance.

During and after the blast, numerous people paid no attention to social distancing rules and went out to streets, hospitals, and crowded areas to check on others, offer support, or just watch. The government got overburdened dealing with the aftermath. This restrained their capacity to oversee and control the implementation of COVID-19 preventive measures.^4^

In reaction to the crisis, the Lebanese Government declared a 2-week state of emergency.

The people who were protesting since 2019 regarding the planned taxes on gasoline, tobacco, and Voice over Internet Protocol calls broke COVID-19 restrictions and went back to the streets again to demonstrate against the unsatisfactory government actions. This caused the prime minister and his cabinet to resign.^5^

In summary, the Beirut explosions have worsened the economic and political situation in Lebanon. They have affected people’s living conditions and health negatively, especially their mental health. It has also played a role in COVID-19 transmission within the community. More focus should be made on community support and engagement on different levels to ensure better trust and involvement in following health guidance. Multisectoral and interprofessional approaches and strategies are essential to move ahead with the current situation. Mental health care can have a crucial effect in improving people’s conditions and attitudes.
